# Effect of combined irradiation and EGFR/Erb-B inhibition with BIBW 2992 on proliferation and tumour cure in cell lines and xenografts

**DOI:** 10.1186/s13014-014-0261-z

**Published:** 2014-12-02

**Authors:** Kristin Gurtner, Nadja Ebert, Dorothee Pfitzmann, Wolfgang Eicheler, Daniel Zips, Michael Baumann, Mechthild Krause

**Affiliations:** Department of Radiation Oncology, UniversityHospital C.G. Carus, Fetscherstr. 74, 01307 Dresden, Germany; OncoRay – National Centerfor Radiation Research in Oncology, Medical Faculty and University Hospital Carl Gustav Carus, TechnischeUniversität and Helmholtz-Zentrum Dresden –Rossendorf, Dresden, Germany; Department of Radiation Oncology, University Hospital Tuebingen, Tuebingen, Germany; German Cancer consortium (DKTK) Dresden and German Cancer Research Center (DKFZ) Heidelberg, Heidelberg, Germany; Helmholtz-Zentrum Dresden - Rossendorf, Dresden, Germany

**Keywords:** Combined treatment, Molecular targeting, EGFR/ErbB-inhibition, Fractionated irradiation, Local tumour control, BIBW 2992

## Abstract

**Background and purpose:**

In previous experiments an enhanced anti-proliterative effect of the EGFR/ErbB tyrosine kinase inhibitor (TKI) BIBW 2992 with single dose irradiation was observed in FaDu tumour xenografts. Aim of the present experiment was to determine if this effect can also be seen in combination with a fractionated radiotherapy. Secondly we investigate the efficacy of BIBW 2992 on local tumour control for UT-SCC-15.

**Material and methods:**

Tumour pieces of FaDu, UT-SCC-14, A431, UT-SCC-15 (squamous cell carcinomas) and A7 (glioma) tumour models were transplanted onto the right hind leg of NMRI (nu/nu) nude mice. For evaluation of tumour growth mice were either treated daily orally with BIBW 2992 (30 mg/kg body weight), or carrier up to a final tumour size of 15 mm or with a fractionated radiotherapy (15f/15d, 30 Gy) with simultaneous application of BIBW 2992 or carrier. For local tumour control UT-SCC-15 tumours were treated with a fractionated radiotherapy (30f/6weeks) or received 30f/6 weeks in combination with daily orally BIBW 2992 (22.5 mg/kg b.w.) during RT.

**Results:**

A significant effect on tumour growth time was observed in all tumour models for BIBW 2992 application alone. However, substantial intertumoural heterogeneity could be seen. In the UT-SCC-14, UT-SCC-15 and A431 tumour models a total regression of the tumours and no recurrence during treatment time (73 days) were determined where as for the A7 tumour only a slight effect was noticeable. For the combined treatment of fractionated radiotherapy (15f/15d) and BIBW 2992 administration a significant effect on tumour growth time was seen compared to irradiation alone for A7, UT-SCC-15 and A431 (ER 1.2 – 3.7), this advantage could not be demonstrated for FaDu and UT-SCC-14. However, the local tumour control was not altered for the UT-SCC-15 tumour model when adding BIBW 2992 to fractionated irradiation (30f/6weeks).

**Conclusion:**

A heterogeneous effect on tumour growth time of BIBW 2992 alone as well as in combination with fractionated irradiation could be demonstrated for all tumour models. However, the significant effect on tumour growth time did not translate into an improvement of local tumour control for the UT-SCC-15 tumour model.

## Introduction

Overexpression of the epidermal growth factor receptor (EGFR) on tumour cells has been shown to increase chemo- and radioresistance and therefore is associated with a poor outcome [1-3]. Inhibition of the EGFR in combination with radiotherapy has become a promising strategy to overcome this resistance. While anti-EGFR antibodies like cetuximab have the potential to prolong tumour growth and improve local tumour control when applied simultaneously to irradiation [4-10], for tyrosine kinase inhibitors (TKI), e.g. Erlotinib, the prolongation of tumour growth time did not translate into improved curative effects [1,8,11]. Clinical evaluation of EGFR-TK inhibition in combination with chemo- or radiotherapy revealed also heterogeneous effects [1,12]. One reason for the rather minor effects of TKI on local tumour control could be that through heterodimerisation with other receptors of the EGFR-family, e.g. ErbB2, signaltransduction is still possible and therefore exclusively blocking the EGFR-TK is not sufficient [13-15]. Targeting more than one receptor of the EGFR-family might therefore show a therapeutic benefit.

BIBW 2992 is an irreversible ErbB-family (EGFR/ErbB2/ErbB4) inhibitor, which in previous experiments demonstrated a significant prolongation of tumour growth time in a combined setting with single dose irradiation in FaDu tumour xenografts. In vitro but not in vivo a radiosensitizing effect could be shown for this tumour model. The antiproliferative effects are in line with a clear G0/G1 arrest of the tumour cells [16]. Based on these findings, the aim of the current study was to investigate the effect of BIBW 2992 with or without fractionated irradiation on tumour growth in five different human tumour xenografts (A7, A431, FaDu, UT-SCC-14 and UT-SCC-15). Consecutively, after these first experiments a local tumour control experiment was performed in the tumour model with the greatest effect on tumour growth time (UT-SCC-15).

## Material and methods

### Animals and tumours

7 to14-week-old male and female NMRI (nu/nu) mice from the specific pathogen-free animal breeding facility of the Experimental Center of the Medical Faculty Carl Gustav Carus, Technische Universität Dresden were used for the experiments. The facility veterinarians checked the microbiological status of the animals regularly. The experiments and animal facilities were approved according to the German animal welfare regulations. Water ad libitum and a commercial laboratory animal diet were provided for the animals. A constant temperature of 26°C, daylight in addition to a 12 h light–12h dark electric cycle (light-on time 07.00 a.m.) and a relative humidity of 50-60% were supplied within the animal room. A whole-body irradiation 2 – 5 days before tumour transplantation with 4 Gy using 200 kV X-rays (0.5 mm Cu) at a dose rate of about 1 Gy/min was given for further immunosuppression of the animals.

Four established human squamous cell carcinoma lines (FaDu, UT-SCC-14, UT-SCC-15, A431) and a glioma cell line (A7) were used in this study. UT-SCC-14 and UT-SCC-15 were established by Prof. Reidar Grenman (University of Turku, Finland). UT-SCC-14 [17,18] was derived from a squamous cell carcinoma of the tongue, UT-SCC-15 [17] is a tumour cell line originating of a recurrent tumour of the tongue. FaDu is an undifferentiated tumour of the hypopharynx [6,17,19-21], originally obtained from the American Type Culture Collection (ATCC) that differs from the original tumour by an additional loss of heterozygosity in the p53 gene (TP53) [22]. A431 is a squamous cell carcinoma of the female genitales (DSMZ, German Collection of Microorganisms and Cell Cultures, Braunschweig, Germany) and A7 is a glioblastoma cell line (Gray Cancer Institute, Mount VernonHospital, Northwood, Middlesex, Great Britain). A cryostock of tumour pieces of all tumour models is kept in Dresden. Out of the cyrostock, generated tumours were passaged in nude mice over 2 generations. The origin of the tumours was validated during passaging by histological examinations, short tandem repeats analysis (microsatellites), and lactate dehydrogenase isoenzyme pattern. Additional histological examinations, evaluations of the volume doubling time and of the lactate dehydrogenase isoenzyme pattern were carried out in parallel to the experiments to ensure constancy of the models and exclude murinization. No or very little residual immune response in nude mice was shown for 4 of the 5 cell lines in previously reported studies [17,23-26]. For A431 tumours local control experiments with and without whole body irradiation were performed revealing no immune response reaction against this tumour line by nude mice. The TCD_50_ for single dose irradiation under clamp conditions with whole body irradiation was 58.6 Gy [95% CI: 60;174] and without whole body irradiation 67.8Gy [95% CI: 51;277] (p = 0.577).

In the experiments, tumour pieces of 1–2 mm diameter were transplanted subcutaneously into the right hind leg of anesthetised (16 mg/kg body weight (b.w.) xylazine [intraperitoneal, i.p.] and 120 mg/kg b.w. ketamine [i.p.]) animals. All procedures for tumour transplantation have been described previously in detail [8,26-28].

Animal welfare approval number: 24D-9168.11-1/2006-20.

### Administration of BIBW 2992

BIBW2992, a second generation tyrosine kinase inhibitor of the epidermal growth factor receptor (EGFR) family binds irreversibly to the intracellular tyrosine kinase of the EGFR and ErbB2 and ErbB4-receptor [29,30]. Its effectiveness *in vitro* and *in vivo* has been shown to be greater than that of the first generation TKIs (e.g. erlotinib) [30] and resistance to first generation EGFR inhibitors could be overcome in certain cell lines by BIBW 2992 [29]. BIBW 2992 was kindly supplied by Boehringer Ingelheim RCV, Vienna Austria.

For evaluation of the drug effect alone, carrier or BIBW 2992 was administered daily orally at a concentration of 30 mg/kg b. w. up to the final size of the tumour (one diameter reaching 15 mm). Within the combined treatment, carrier or BIBW 2992 were only given simultaneously during fractionated irradiation using the same application and concentration schedule mentioned above, with a 4 hour interval before each irradiation fraction. For the local control experiment a lower BIBW 2992 concentration (22.5 mg/kg b.w.) was administered due to observed toxicity within the previous experiments.

### Local tumour irradiation

Local tumour irradiation was carried out under ambient conditions to air-breathing animals without anaesthesia (200 kV X-rays, 0.5 mm Cu, single beam, dose rate ~1 Gy/min, source to skin distance 42 cm). Specially designed jigs were able to hold 5 animals for simultaneous irradiation. The tumour-bearing leg was held positioned in the irradiation field while mice were immobilized in a plastic tube which was fixed on a lucite plate by a foot-holder distal to the tumour.

### Experimental design

**Figure 1:**
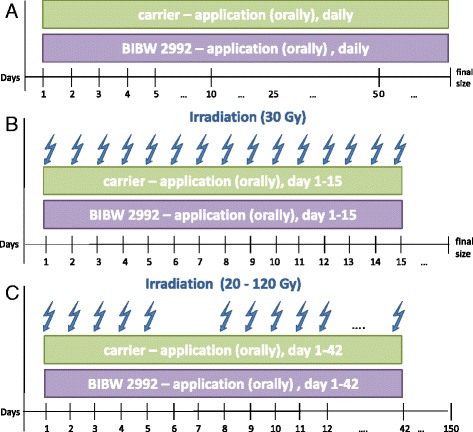
**Experimental design. A)** Application of either carrier or BIBW 2992 up to the final size of the tumour. **B)** Fractionated irradiation (15f; total dose 30 Gy) in combination with carrier or BIBW 2992 during irradiation time. **C)** Fractionated irradiation (30f/6 weeks/total doses between 20 and 120 Gy) in combination with carrier or BIBW 2992 during irradiation time.

The first experiment was divided into 2 arms (Figure 1): in arm (A) animals were treated with either carrier or BIBW 2992 orally daily up to the final size of the tumour (14–16 animals per group). In the second arm (B) tumours were additionally irradiated with 15 fractions applying one fraction per day (14–16 animals per group). Carrier or BIBW 2992 were given 4 hours before each irradiation fraction without continuation after the end of irradiation.

**Figure 2:**
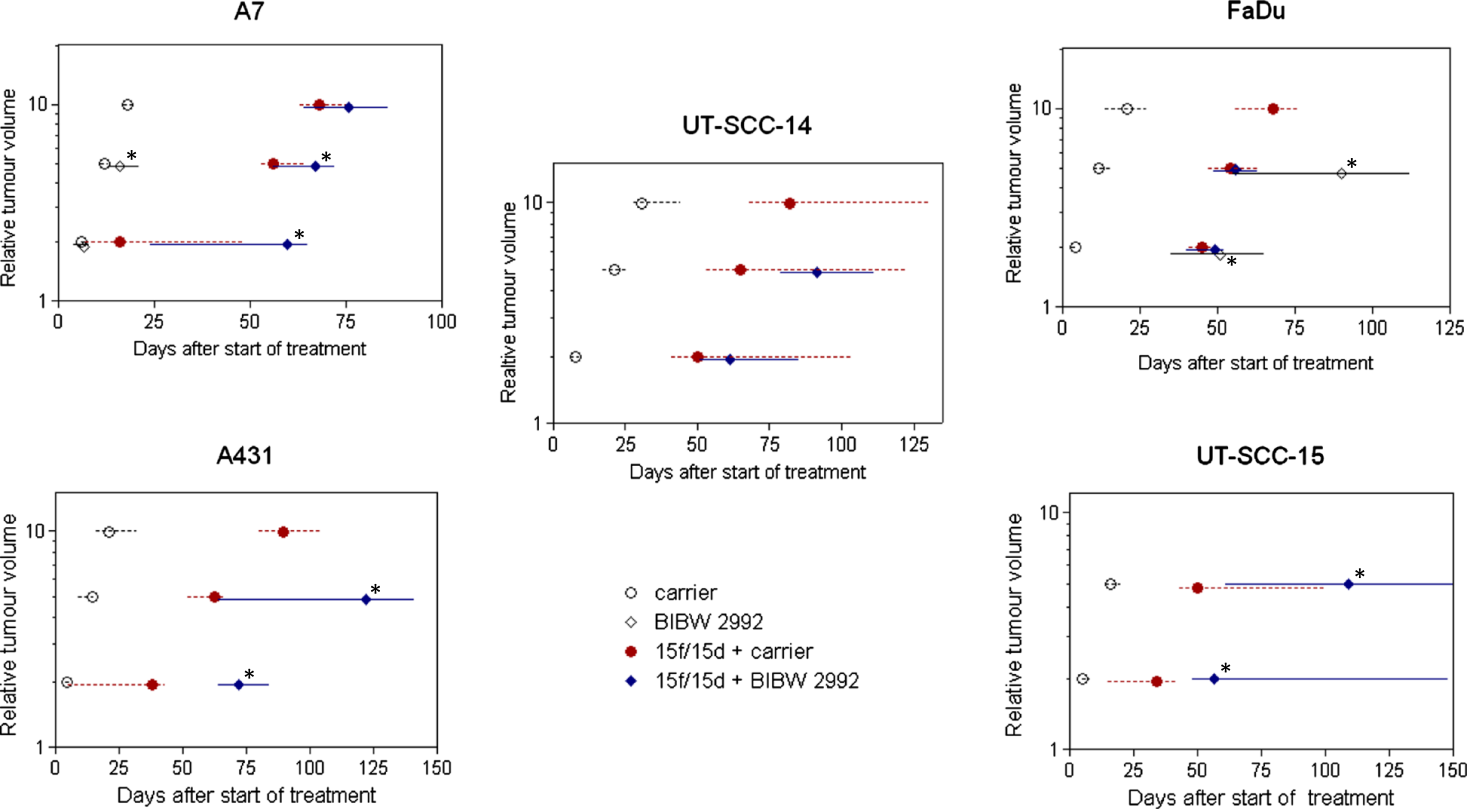
**Effect on tumour growth time.** Time to reach 2-fold or 5-fold the starting volume for A7, A431, FaDu, UT-SCC-14 and UT-SCC-15 xenografts receiving either carrier (○) or BIBW 2992 (◊) or the combined treatment of 15f/15d + carrier (closed circle symbol) or 15f/15d + BIBW 2992 (closed diamond symbol). Symbols represent median and bars 95% confidence intervals. p-value in comparison to control groups *significant difference (comparison between BIBW 2992 vs. carrier or combined IR + BIBW 2992 vs. IR + carrier).

For the local tumour control experiment (C), the UT-SCC-15 tumour model was selected as the best responding model regarding tumour growth time (Figure 2). UT-SCC-15 tumours were irradiated with 30 fractions within 6 weeks up to total irradiation doses of 20 to 120 Gy (9 dose groups, 6–8 animals per dose group). As in the first experiment, carrier or BIBW 2992 were applied 4 hours before each irradiation fraction and continued over the irradiation-free weekends, but not after the end of irradiation.

Tumour volumes for both experiments at start of treatments were approximately 100 mm^3^ (median volume 112 mm^3^, 10-90% = 100-160 mm^3^).

### Follow-up

Animals were observed up to final tumour size (mean diameter exceeded 15 mm), until day 150 after the end of treatment or until death. For UT-SCC-15 tumours that were evaluated for local tumour control, it has been shown before that almost all recurrences occur within that follow-up time after radiotherapy [17]. In the local control experiment 96% of 46 recurrences after irradiation occurred before day 126, the last recurrence was seen at day 130. Animals that appeared to suffer needed to be killed before these endpoints were reached.

### Determinations of tumour volume and tumour growth time

Tumour diameters were determined twice per week. With the formula for the rotational ellipsoid V = π/6 * a * b^2^, where a is the longest and b is the perpendicular shorter tumour axis, tumour volumes were calculated. For each treatment arm the median tumour volumes and their standard errors were computed as a function of time after start of treatment. Evaluations were stopped when < 50% of the animals of each treatment arm were alive. From individual growth curves the median tumour growth time was calculated as the time that a regrowing tumour needed to reach 2 and 5 fold of the starting volume (GD_V2,_ GD_V5_). Enhancement ratios (ER) were computed as the quotient of a GD value of BIBW 2992 treated tumours and the GD value in the carrier treated group.

### Dose–response curves for local tumour control and TCD_50_ values

150 days after end of irradiation tumour control rates were calculated for each dose group after correction for censored animals according to the method given by Walker and Suit [31]. When an increased volume for at least three consecutive measurements was observed after passing a nadir, recurrences were scored. Animals that died from tumour-independent reasons and therefore could not be followed up, were censored at the last day of measurement.When death occurred before day 20 after end of treatment animals (without recurred tumour) were excluded from the analysis. Animals with local failure before death were not excluded from the analysis but counted as failure. Based on the individual tumour control data a binary (cure/failure) model was used to fit tumour control probability (TCP) curves. The TCP was modeled using the logit model

$$ \mathrm{T}\mathrm{C}\mathrm{P} = 1/\left[1 + \exp\ \left(-f\Big(x,\beta \right)\right] $$

where *x* = vector of covariates that define the treatment, *β* = vector of parameters describing radiosensitivity of the tumours, and *f* is a (possibly nonlinear) function of these. Parameters were estimated using maximum likelihood as implemented in STATA 7.0 software (STATA Corporation, College Station TX). Quoted confidence limits are asymptotic estimates from the results of the likelihood fits. Comparison of maximum likelihood fits was performed using the likelihood ratio test. Tumour control dose 50% (TCD_50_) at day 150 after end of irradiation and associated dose–response curve were determined from:

$$ f\left(D,\beta \right)={\beta}_1\left(1-D/{\beta}_2\right)\kern0.37em \mathrm{where}\ {\beta}_1\kern0.5em \mathrm{is}\ \mathrm{a}\ \mathrm{constant}\ \mathrm{a}\mathrm{nd}\ {\mathrm{TCD}}_{50}={\beta}_{2.} $$

### Cell survival in vitro

Tumour cells of the different cell lines were grown in Dulbeccos modified Eagle’s medium with glutamine, 10% fetal calf serum, 1 mM pyruvate, 1% non-essential amino acids, 20 mM HEPES and 1% Penicillin-Streptomycin at 37°C (5% CO_2_, 95% humidity) in 25 cm^2^ tissue culture flasks. For the A7 tumour model MEM-EARLE with 10% fetal calf serum and 1 mM pyruvate was used as medium. After 24 hours cells were incubated with BIBW 2992 or for control with dimethylsulfoxid (DMSO). Three days later, cells were irradiated with doses of 2, 4, 6 or 8 Gy. Directly after irradiation (200 kV x-rays, 0.5 mm Cu, ~1 Gy/min) cells were trypsinised and counted. Appropriately diluted single cell suspensions were incubated in petri dishes for 14 or 10 days (A431), fixed and stained with crystal violet.Colonies with ≥ 50 cells were scored as survivors. Mean values of the surviving fraction and their standard deviations (SD) were determined for each treatment group and dose level. Cell survival curves were fitted according to the LQ-model. Plating efficiencies (PE) and surviving fractions (SF) were calculated using the following formulas:

$$ \mathrm{P}\mathrm{E} = \left(\mathrm{colonies}/\mathrm{plated}\ \mathrm{cells}\right)*100\%;\ \mathrm{S}\mathrm{F} = \left(\mathrm{P}\mathrm{E}\ \mathrm{of}\ \mathrm{each}\ \mathrm{dose}/\ \mathrm{P}\mathrm{E}\ 0\ \mathrm{Gy}\right)*100\%. $$

## Results

Figure 2 shows the relative tumour volume as a function of time after start of treatment for all 5 tumour models. For each of the 4 different treatment arms growth time is presented as the time for tumours to reach the 2-fold (GDV_2_) and 5-fold (GDV_5_) of the starting volume. The application of BIBW 2992 alone leads to a significant prolongation of tumour growth time in all tumour models with considerable intertumoural heterogeneity. While for the A7 tumour model only a slight prolongation of tumour growth can be seen, a distinct effect was observed for the FaDu-, A431-, UT-SCC-14 and UT-SCC-15 tumour models. A complete regression of all tumours during continuous application of BIBW 2992 alone without recurrence seen during treatment time (mean 73 days) was determined in the UT-SCC-14, A431 and UT-SCC-15 tumours. During fractionated irradiation (15 fractions within 15 days, total dose 30 Gy) a significant prolongation of tumour growth by BIBW 2992 could be observed for the A7, A431 and UT-SCC-15 tumour models where as this effect was not evident in FaDu and UT-SCC-14 tumours (Table 1). The larger growth inhibiting effect of BIBW 2992 alone compared to the combination with fractionated irradiation can be seen as a consequence of the shorter administration within the combined treatment schedule.

**Table 1:** **Time to reach 2-fold or 5-fold of the starting volume for the five different tumour models and 4 different treatment arms**

	**Carrier**	**BIBW 2992**	**15f/15d + carrier**	**15f/15d + BIBW 2992**
**A7 -Glioblastoma**				
GD_V2_(95% C.I.)	6 d (6; 7)	6.5 d (4; 8)	16 d (7; 48)	59.5 d (24; 65)
ER/p-value	*1.08/1.00*		*3.72/0.01*	
GD_V5_(95% C.I.)	12 d (11; 13)	16 d (13; 21)	56 d (53; 64)	67 d (56; 72)
ER/p-value	*1.33/0.01*		*1.20/0.02*	
**FaDu–SCC from the head and neck**				
GD_V2_(95% C.I.)	4 d (3; 5)	51 d (35; 65)	45 d (41; 48)	49 d (40; 52)
ER/p-value	*12.75/<0.0001*		*1.09/0.63*	
GD_V5_(95% C.I.)	11.5 d (10; 16)	90 d (55;112)	54 d (47; 64)	56 d (49; 63)
ER/p-value	*7.83/<0.0001*		*1.04/0.87*	
**UT-SCC-14 – SCC from the head and neck**				
GD_V2_(95% C.I.)	7.5 d (6; 9)	n. a.	50 d (41; 103)	61 d (51; 85)
ER/p-value	*n. a.*		*1.22/0.30*	
GD_V5_(95% C.I.)	21 d (17; 26)	n. a.	65 d (53; 122)	91.5 d (79; 111)
ER/p-value	*n. a.*		*1.41/0.15*	
**A431–SCC from the cervix**				
GD_V2_(95% C.I.)	4.5 d (4; 7)	n. a.	38 d (5; 43)	72 d (64; 84)
ER/p-value	*n. a.*		*1.89/<0.0001*	
GD_V5_(95% C.I.)	14.5 d (9; 15)	n. a.	62.5 d (52; 66)	122 d (64; 141)
ER/p-value	*n. a.*		*1.95/0.0050*	
**UT-SCC-15 –SCC from the head and neck**				
GD_V2_(95% C.I.)	5 d (5; 8)	n. a.	34 d (15; 42)	56.5 d (48; 148)
ER/p-value	*n. a.*		*1.66/0.03*	
GD_V5_(95% C.I.)	16 d (14; 20)	n. a.	50 d (43; 100)	109 d (61; 182)
ER/p-value	*n. a.*		*2.18/0.04*	

ER and p-values in comparison to control groups. *n.a. – not applicable (tumours did not reach these endpoints).*

**Figure 3:**
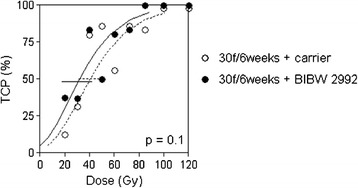
**Observed local tumour control rates (symbols) and calculated local tumour control probabilities for UT-SCC-15 tumours treated with 30 fractions in 6 weeks.** Simultaneously to fractionated irradiation animals received carrier (o, dotted line) or BIBW 2992 (•, solid line). Error bars represent 95% C.I. of the TCD_50_ values.

Figure 3 depicts the dose response curves for the local tumour control experiment in UT-SCC-15 tumours after fractionated irradiation (30 fractions within 6 weeks) with or without simultaneous application of BIBW 2992. The TCD_50_ is not significantly altered when BIBW 2992 is added to fractionated irradiation. The TCD_50_ for irradiation alone is 40.7 Gy [95% CI: 32;51] and for the simultaneous BIBW 2992 administration 32.2 Gy [18;42] (p = 0.104).

**Figure 4:**
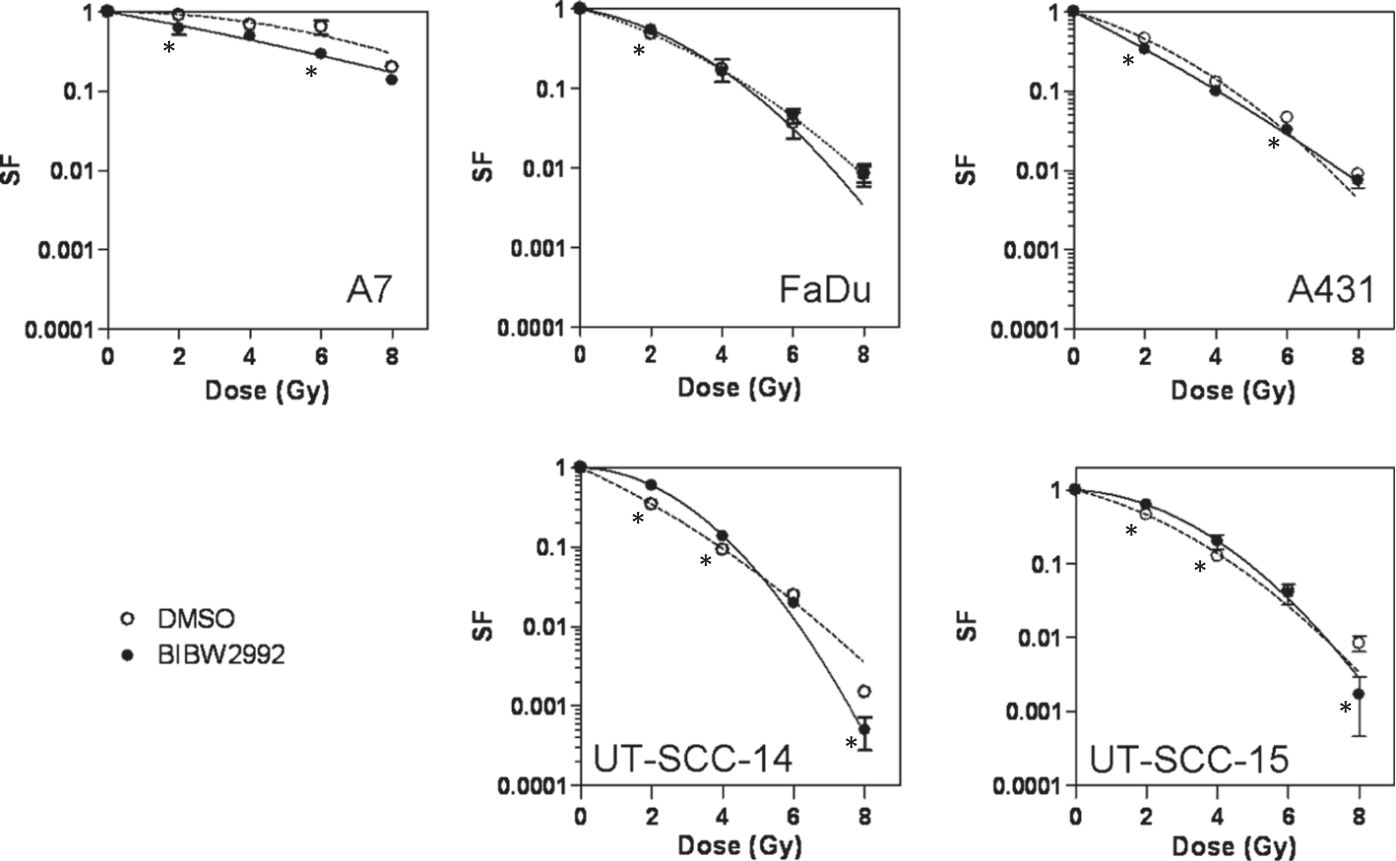
**Clonogenic cell survival of A7, A431, FaDu, UT-SCC-14 and UT-SCC-15 cells irradiated with different total doses after three days incubation with normal medium (DMSO; o) or BIBW 2992 (•), plating directly after irradiation.** Symbols and error bars represent means and standard deviations of three independent experiments (duplicates). The data were fitted according to the LQ model (dotted line = normal medium, solid line = BIBW 2992).* significant difference (for dose groups in comparison to control group).

Figure 4 illustrates the cell survival curves in vitro for the different cell lines. For the tumour models FaDu, A431, UT-SCC-14 and UT-SCC-15 no radiosensitizing effect by incubation with BIBW 2992 could be detected. Only in the A7 glioblastoma cells a slight and for the dose group of 2 and 6 Gy significant radiosenitization could be seen (p-value for 2 Gy = 0.050; p-value for 6 Gy =0.046).

Table 2 shows the heterogeneity of the cytotoxic effect of BIBW 2992 on clonogenic tumour cells (plating efficiency, PE) between the 5 cell lines. While there is no effect of incubation with BIBW 2992 on the clonogenicity of A7-, FaDu- and A431 cells, a significant reduction of clonogenic cell survival could be observed for the UT-SCC-14, UT-SCC-15 cells.

**Table 2:** **Plating efficiency** ***in vitro*** **after incubation with either normal medium or BIBW 2992 for the 5 different cell lines**

**Tumor**	**A7**	**FaDu**	**UT-SCC-14**	**A431**	**UT-SCC-15**
**0 Gy DMSO (%)**	5.76	19.49	5.70	30.31	9.99
**0 Gy BIBW 2992 (%)**	5.70	22.14	3.46	23.99	5.88
**ER/p-Wert**	1.01/0.9733	0.88/0.4274	1.65/0.0463	1.26/0.2180	1.70/0.0005

Enhancement Ratio (ER) calculated as quotient of PE_normal medium_ and PE_BIBW 2992_ for each cell line.

## Discussion

The present experiments are the first to test the effect of combined fractionated irradiation and an EGFR/ErbB-TK inhibitor on tumour growth time and local tumour control. 4 different squamous cell carcinoma cell lines with heterogeneous radiosensitivity and a radioresistant glioma cell line were selected. We could show a heterogeneous effect on tumour growth time in these 5 different tumour xenografts tested for the administration of BIBW 2992 alone or in combination with fractionated irradiation. These findings are in line with previous experiments, where a prolongation of tumour growth time was seen for the treatment of BIBW 2992 with or without single dose irradiation in the FaDu tumour model [16] or bladder tumour model [32]. Li et al. [29] also showed an antiproliferative effect on A431 tumour xenografts by a daily oral application of BIBW 2992 alone [29]. The major intertumoural heterogeneity of the antiproliferative effect is in line with previous experience on the selective EGFR-TK inhibitor erlotinib in 5 different tumour models [8]. With the limitations of a comparison between different experiments, one could conclude that the dual inhibition of EGFR and ErbB-receptor shows no larger effect on the prolongation of tumour growth in the three models treated in both experiments compared to the EGFR-TK inhibitor erlotinib. For UT-SCC-14 and −15 an infinite prolongation with erlotinib or BIBW 2992 could be observed in both experiments. For FaDu, the ER for application of erlotinib was 1.5 for GD_V5_ and for BIBW 2992 an ER of 7.8 for the same endpoint was determined [8]. The latter appears at the first glance as a difference and an advantage for the EGFR-TK inhibitor, but this may well be artificially impacted by the treatment within different experiments.

Local tumour control was evaluated in UT-SCC-15, the cell line with the best response on clonogenic survival after incubation with BIBW 2992 alone *in vitro* and with the largest effect of the combined treatment on tumour growth *in vivo*. The A7 cell line showed also a minimal but significant radiosensitizing effect *in vitro*. However, because of the small amount of this effect and the lower response of this cell line regarding the other endpoints, this was not further followed up. Regarding the effect on local tumour control in UT-SCC-15, there was no benefit seen by the simultaneous ErbB family inhibition with BIBW 2992 during fractionated irradiation (Figure 3). The TCD_50_ was not significantly different between fractionated irradiation alone or combined with BIBW 2992. The local tumour control data are comparable to previous experiences where an inhibition of tumour growth by EGFR-TK inhibitors did not translate into an improvement on local tumour control in different tumour models [1,8,28,33].

Animals receiving BIBW 2992 with or without radiation tolerated the drug well (body weight measured weekly as indicator of well being). Common side effects were erythema of the mouth and diarrhea as described before by Schütze et al. [16]. After an application period of approximately 73 days within the first experiment animals started to lose body weight and died or needed to be sacrificed due to reduced general condition. Therefore we reduced the BIBW 2992 dose for the local tumour control experiment to 22.5 mg/kg b.w.. For early clinical studies on systemic treatment alone (without radiotherapy) the same side effects (erythema and diarrhea) have been observed [34-36] and resemble experiences with other TK-inhibitors like gefitinib or erlotinib [37,38].

It can only be speculated which reasons underly the missing translation of the positive effect on tumour growth into improvement of local tumour control for TK-inhibitors in comparison to antibodies like for example cetuximab. One potential reason could still be an alternative signal transduction. Hu et al. [39] reported a cross talk of the EGFR with the insulin-like growth factor receptor (IGF1R) [39]. Incubating tumour cells with gefitinib or erlotinib led to an increased heterodimerisation of IGF1R and EGFR resulting in a pronounced IGF1R-activation and therefore amplified activation of downstream mediators [1,40,41]. In these *in-vitro* experiments, the induced resistance to EGFR-TKI could be overcome by IGF1R-inhibitors [1,40,41]. Independent on the biological reasons, the differential effect of BIBW 2992 on tumour proliferation versus local tumour control is finally caused by a missing net effect on cancer stem cells. This is based on the knowledge that all cancer stem cells need to be eliminated to cure a tumour and that a single surviving cancer stem cell after treatment can cause a recurrence, thus making tumour control experiments to an indirect measure of CSC survival in situ [42]. It is interesting to note that BIBW 2992 *in vitro* does inactivate clonogenic cells in UT-SCC-15 and UT-SCC-14 independent from the irradiation effect (Table 2). If this independent clonogenic cell kill would translate into an inactivation of cancer stem cells in vivo, one would have expected an improvement of local tumour control by the combined treatment. Reasons for the missing translation might be that BIBW 2992 and irradiation preferentially target the same tumour cell population, thus diluting the cytotoxic effect of BIBW 2992 when combining these treatments.

## Conclusion

The present experiments show a heterogeneous effect of the ErbB family TK inhibitor BIBW 2992 on tumour growth in 5 different tumour models for drug application alone as well as in combination with a fractionated radiotherapy. The major effect on tumour growth in UT-SCC-15 tumours did not translate into an improvement of local tumour control. Along with previous experiments on combined fractionated irradiation and EGFR-TK inhibition, it appears likely that TKI do not affect survival of tumour cells that can cause recurrences but can lead to a good palliative effect by proloning tumour growth for a reasonable amount of time.
